# The MRI posterior drawer test to assess posterior cruciate ligament functionality and knee joint laxity

**DOI:** 10.1038/s41598-021-99216-w

**Published:** 2021-10-04

**Authors:** Lena Marie Wollschläger, Karl Ludger Radke, Justus Schock, Niklas Kotowski, David Latz, Dominika Kanschik, Timm Joachim Filler, Svenja Caspers, Gerald Antoch, Joachim Windolf, Daniel Benjamin Abrar, Sven Nebelung

**Affiliations:** 1 Department of Diagnostic and Interventional Radiology, Medical Faculty, University Dusseldorf, Moorenstraße 5, 40225 Düsseldorf, Germany; 2 Department of Orthopaedics and Trauma Surgery, Medical Faculty, University Dusseldorf, 40225 Düsseldorf, Germany; 3 Department of Diagnostic and Interventional Radiology, Aachen University Hospital, Aachen, Germany; 4 Institute of Anatomy I, Heinrich-Heine-University, 40225 Düsseldorf, Germany

**Keywords:** Anatomy, Biomarkers, Medical research, Imaging, Musculoskeletal system

## Abstract

Clinical Magnetic Resonance Imaging (MRI) of joints is limited to mere morphologic evaluation and fails to directly visualize joint or ligament function. In this controlled laboratory study, we show that knee joint functionality may be quantified in situ and as a function of graded posterior cruciate ligament (PCL)-deficiency by combining MRI and standardized loading. 11 human knee joints underwent MRI under standardized posterior loading in the unloaded and loaded (147 N) configurations and in the intact, partially, and completely PCL-injured conditions. For each specimen, configuration, and condition, 3D joint models were implemented to analyse joint kinematics based on 3D Euclidean vectors and their projections on the Cartesian planes. Manual 2D measurements served as reference. With increasing PCL deficiency, vector projections increased significantly in the anteroposterior dimension under loading and manual measurements demonstrated similar patterns of change. Consequently, if combined with advanced image post-processing, stress MRI is a powerful diagnostic adjunct to evaluate ligament functionality and joint laxity in multiple dimensions and may have a role in differentiating PCL injury patterns, therapeutic decision-making, and treatment monitoring.

## Introduction

The posterior cruciate ligament (PCL) is an essential stabilizer of the knee joint and prevents excessive posterior tibial translation (PTT) and external rotation^3,21,28^. Injuries to the PCL are caused by excessive forces on the tibia, by hyperflexion or by hyperextension of the joint^39^. Due to secondary biomechanical alterations of the PCL-deficient joint, missed PCL injuries are likely to induce progressive joint surface damage and, ultimately, post-traumatic osteoarthritis^4,6,23^. Timely and accurate diagnosis of PCL injuries is therefore crucial for successful treatment and prevention of joint instability and irreversible damage^25^.

The *posterior drawer test* is a commonly applied clinical test for diagnosing PCL injuries with excellent sensitivity and specificity rates of 90–99%^10,34^. The extent of PTT directly affects surgical decision-making: While patients with PTT values ≥ 10 mm should undergo surgery, those with PTT values < 10 mm may be treated conservatively^14,30,38^. Previously injured and scarred PCLs with compromised function as well as acute partial PCL injuries may bring about false negative clinical findings^9,33,39^, thereby challenging and delaying establishment of correct diagnoses^25^.

The diagnosis of complete, acute PCL injuries via magnetic resonance imaging (MRI) is characterized by excellent sensitivity and specificity rates of 97–100%^5,20,21^ and often unequivocally established by total fiber disruption^30^. In contrast, partial PCL injuries may appear normal on MRI^13,33,40^, thus challenging accurate diagnosis^8,28,39^. Consequently, MRI performs poorly with sensitivity rates of 62–67% when it comes to differentiating partial from complete PCL injuries^9,32^. This diagnostic limitation is due to the fact that injured and functionally insufficient PCLs appear morphologically largely intact, i.e., continuous and homogeneously hypointense because of constitutive healing processes^1,8,33^. Chronic and partial intra-substance PCL injuries may thus be missed as only subtle findings such as apparent thickening or increased signal intensity may indicate injury to the ligament^1,32,33^. Moreover, variable injury patterns and imaging characteristics further challenge distinction of partial from complete PCL injuries, intact ligaments or mucoid degeneration^31,39^. Most likely, unphysiological imaging conditions (with the patient supine and the joint unloaded) are at the root of these limitations and prevent full exploitation of the technical and diagnostic potential of MRI^1,9,39^.

With morphologic continuity potentially masking PCL injuries on MRI, stress radiography has become the diagnostic cornerstone of functional PCL assessment and is strongly advocated in suspected chronic PCL tears^29^. Stress radiography may quantify PTT and posterior knee laxity, thereby aiding in subsequent therapeutic decision-making^15,16,21^. However, utilization of ionizing radiation, limited 2D visualization, low-to-moderate reproducibility and accuracy, and insufficient depiction of soft tissues are substantial limitations and caveats in clinical practice^16,22^.

Against this background, the present study aimed (1) to establish the *MRI posterior drawer test* to simultaneously assess PCL status and knee laxity and (2) to apply it in an arthroscopic *in-situ* model of graded PCL injury. Our hypotheses were that (1) MRI techniques, complemented with loading, can be used to quantify the degree of PCL injury in multiple dimensions to differentiate intact PCLs from partial and complete PCL injuries and that (2) advanced image post-processing techniques are as accurate as manual reference measurements in quantifying joint laxity.

## Results

All 11 specimens underwent complete MR imaging in all PCL conditions, i.e., intact (PCL_intact_), partially PCL-deficient (PCL_partial_), and completely PCL-deficient (PCL_complete_), and in all configurations, i.e., unloaded (δ_0_) and loaded (δ_1_).

**Figure 1:**
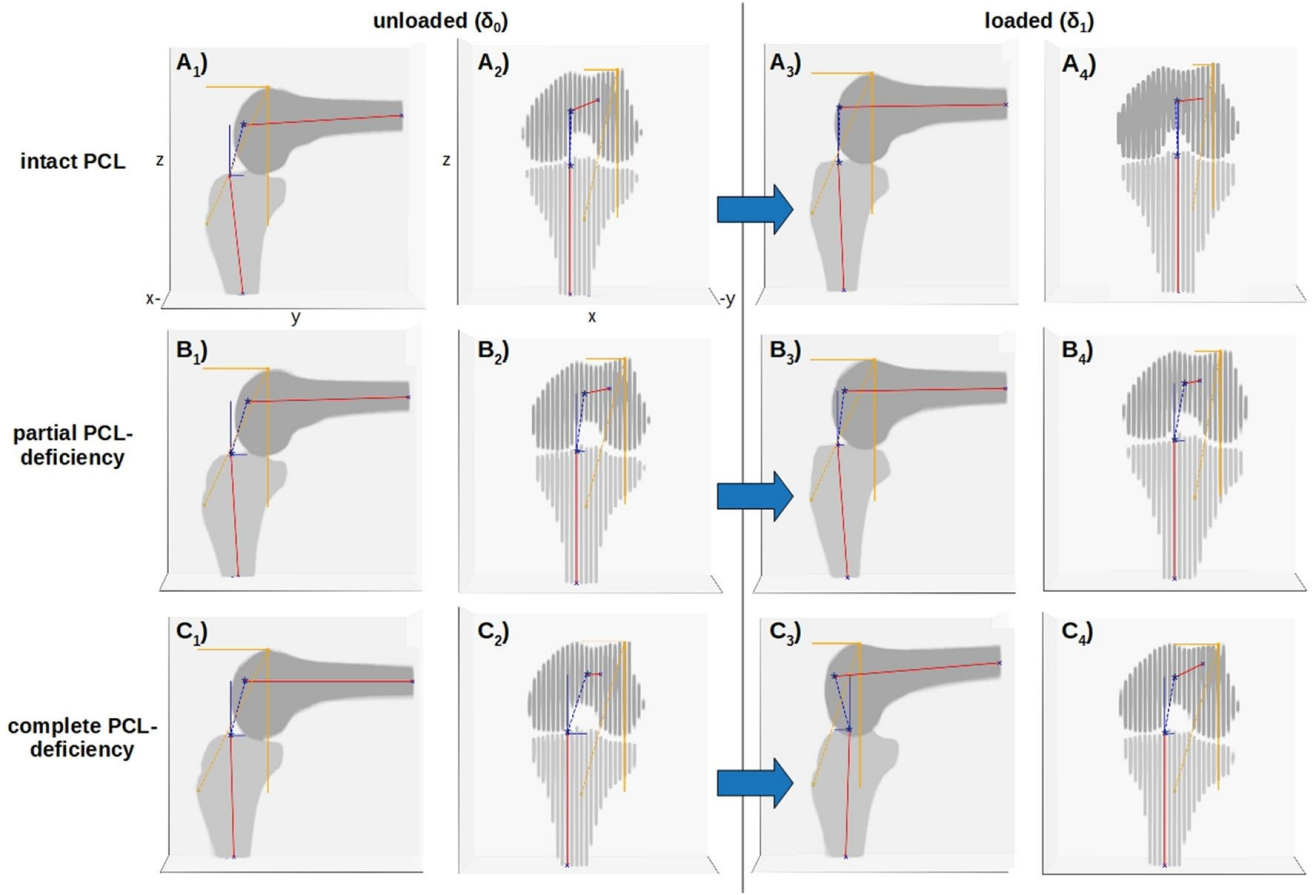
Multidimensional 3D Computed Vector Measures to Quantify Joint Laxity as a Function of PCL Injury and Loading. Displayed are the PCL-intact (**A**), partially (**B**), and completely (**C**) PCL-deficient conditions in the unloaded (**A**_**1–2**_–**C**_**1–2**_) and loaded (**A**_**3–4**_–**C**_**3–4**_) configurations of a representative left knee joint specimen. Manually segmented bone contours of the femur (dark grey) and tibia (light grey) are displayed in the yz- (sagittal, **A**_**1**_–**C**_**1**_, **A**_**3**_–**C**_**3**_) and xz-dimensions (coronal, **A**_**2**_–**C**_**2**_, **A**_**4**_–**C**_**4**_). While the amount of posterior translation of the tibia relative to the femur was limited in the PCL-intact (**A**) and partially PCL-deficient conditions (**B**), it was considerably larger in the presence of complete PCL injury (**C**). Blue block arrows indicate the direction of force during the *MRI posterior drawer test*. Please refer to Supplementary Fig. 1 and the main text for details on vectors, axes, fixpoints, and anatomic landmarks. Same knee joint specimen as in Fig. 2 and Supplementary Figs. 1 and 2.

**Figure 2:**
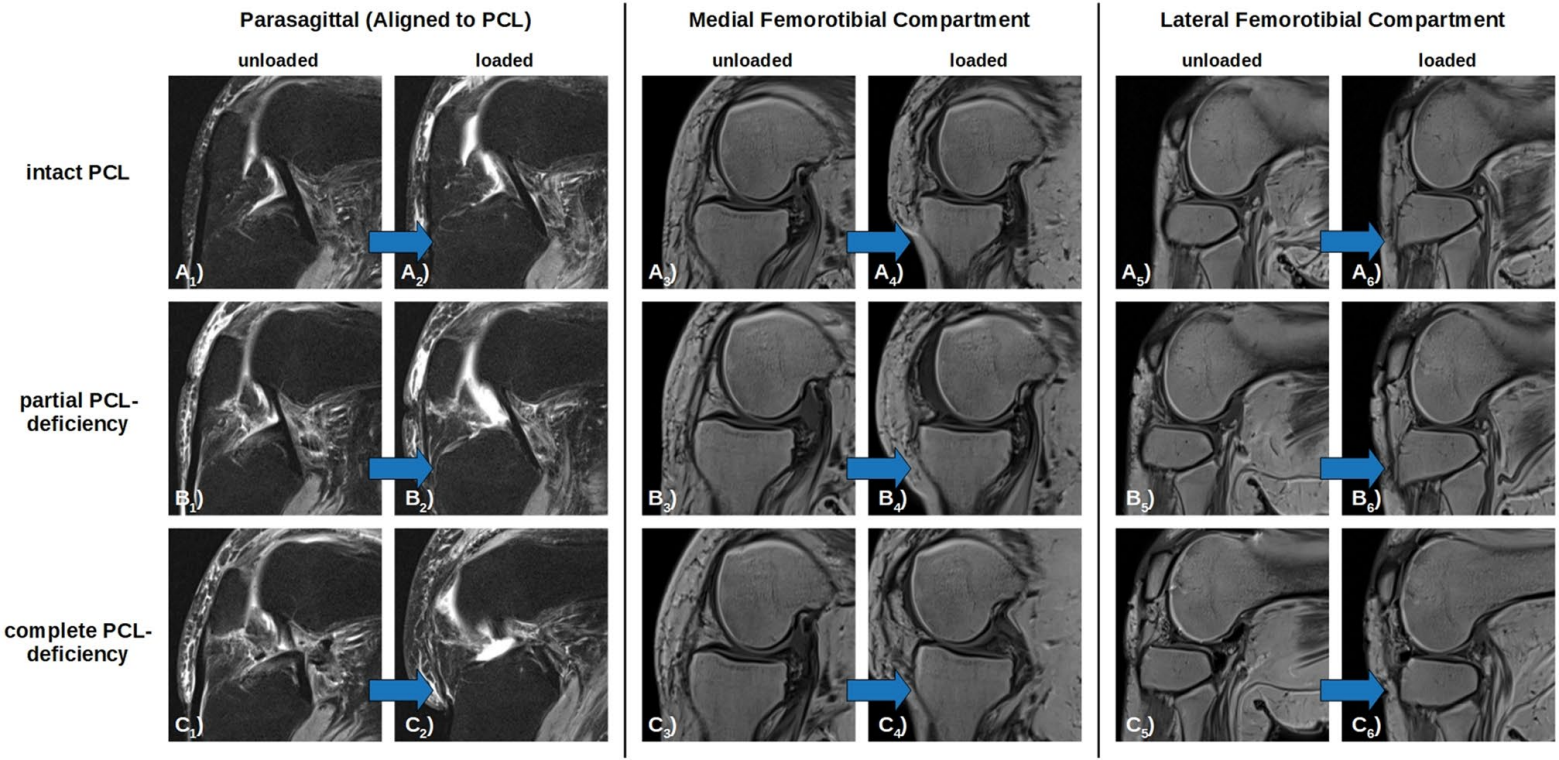
Loading-Induced Changes in the Knee Joint as a Function of PCL Injury and Loading. Displayed are the unloaded (**A**_**1**_–**C**_**1**_, **A**_**3**_–**C**_**3**_, **A**_**5**_–**C**_**5**_) and loaded (**A**_**2**_–**C**_**2**_, **A**_**4**_–**C**_**4**_, **A**_**6**_–**C**_**6**_) configurations of the PCL-intact (**A**), partially PCL-deficient (**B**), and completely PCL-deficient (**C**) conditions. Images were acquired along the course of the PCL (parasagittal, PD-weighted fat-saturated sequence [**A**_**1**_–**C**_**1**_, **A**_**2**_–**C**_**2**_]) and along the central medial (**A**_**3**_–**C**_**3**_, **A**_**4**_–**C**_**4**_) and lateral (**A**_**5**_–**C**_**5**_, **A**_**6**_–**C**_**6**_) femorotibial compartments (sagittal, T1-weighted sequences). Blue block arrows indicate the direction of loading. Posterior tibial translation increased as a function of loading and PCL injury and was larger in the lateral than medial femorotibial compartment. Same left knee joint specimen as in Fig. 1 and Supplementary Figs. 1 and 2.

Complex joint changes were observed as a function of PCL condition and loading. In the PCL_intact_ and PCL_partial_ conditions (Fig. 1A, B), loading-induced PTT was limited, while it was considerably larger in the PCL_complete_ condition (Fig. 1C). PTT was largest in the PCL_complete_ condition, in the δ_1_-configuration, and in the lateral femorotibial compartment (Fig. 2).

For the 3D computed vector measures (Table 1) and 2D manual reference measures (Table 2), absolute differences versus the unloaded PCL_intact_ condition are detailed as a function of condition and configuration. The corresponding post-hoc test results are given in Supplementary Tables 1 and 2, while the absolute values of each measure are presented in Supplementary Table 3.

**Table 1:** Absolute differences of 3D vector measures to compute knee joint changes as a function of PCL injury and loading.

	Measure (mm)	Absolute differences Δ (vs. PCL_intact_ [δ_0_])					*p*-value
		PCL_intact_	PCL_partial_		PCL_complete_		
		Δ_1_	Δ_0_	Δ_1_	Δ_0_	Δ_1_	
Axis-surface-intersections	vector_ASI	− 4.2 ± 5.2	− 2.0 ± 3.2	− 2.0 ± 5.0	− 1.7 ± 3.1	− 2.6 ± 3.5	0.415
	x_ASI	− 1.7 ± 8.6	0.3 ± 8.1	1.1 ± 6.7	4.4 ± 4.4	− 0.6 ± 7.8	0.181
	y_ASI	7.9 ± 3.2	1.6 ± 5.8	9.8 ± 5.8	0.5 ± 4.0	15.2 ± 5.1	< 0.001^1^
	z_ASI	− 3.3 ± 4.7	− 1.6 ± 2.8	− 0.8 ± 4.6	− 1.7 ± 2.2	− 2.1 ± 2.8	0.457
Anatomic landmarks	vector_FT	− 3.4 ± 2.8	0.2 ± 7.5	− 1.2 ± 11.0	2.4 ± 4.0	− 4.9 ± 3.0	0.101
	x_FT	− 5.4 ± 7.0	1.5 ± 6.0	− 2.1 ± 6.8	2.1 ± 5.4	− 3.6 ± 10.1	0.008^2^
	y_FT	6.6 ± 2.4	− 1.2 ± 5.0	4.4 ± 5.7	− 1.3 ± 5.1	12.0 ± 5.0	< 0.001^3^
	z_FT	0.0 ± 3.7	− 0.5 ± 7.5	1.1 ± 9.8	1.7 ± 3.7	− 0.1 ± 3.5	0.894

For all 3D computed vector measures, absolute values for each PCL-condition (PCL_intact_—intact PCL; PCL_partial_—after partial PCL transection; PCL_complete_—after complete PCL transection) and loading configuration (unloaded [δ_0_] and loaded [δ_1_]) were related to absolute values of δ_0_ [PCL_intact_] to determine absolute changes (Δ_x_) for each PCL condition (Δ_x_[PCL-condition] = δ_x_[PCL-condition]-δ_0_[PCL_intact_]). Data are presented as means ± standard deviation. Statistical analysis was based on repeated measures analysis-of-variance and significant findings are indicated in bold type. Sequential numbers in square brackets indicate the corresponding post-hoc details as given in Supplementary Table 1. For detailed explanations of the vector-based 3D measurements, please refer to Supplementary Fig. 1. Abbreviations: PCL—posterior cruciate ligament. Vector_ASI—vector between the femoral and the tibial axis-surface-intersection. Vector_FT—vector between the apex of the femoral trochlea and the centre of the tibial tuberosity. Vector projections on Cartesian x-axis (i.e., anteroposterior dimension; “x_FT”, “x_ASI”), y-axis (i.e., mediolateral dimension; “y_FT”, “y_ASI”), and z-axis (i.e., craniocaudal dimension; “z_FT”, “z_ASI”) are given, too.

**Table 2:** Absolute differences of 2D manual reference measures as a function of PCL injury and loading.

	Femorotibial Compartment	Measure [mm]	absolute differences Δ (vs. PCL_intact_ [δ_0_])					*p*-value
			PCL_intact_	PCL_partial_		PCL_complete_		
			Δ_1_	Δ_0_	Δ_1_	Δ_0_	Δ_1_	
Reader 1	Medial	AD-MM	− 0.5 ± 2.5	1.6 ± 3.5	− 0.5 ± 2.6	1.3 ± 5.4	− 4.7 ± 3.0	< 0.001^4^
		PD-MM	− 1.1 ± 3.2	1.0 ± 2.1	− 0.5 ± 3.3	− 0.1 ± 4.2	− 5.5 ± 5.5	< 0.001^5^
		API-MM	− 1.0 ± 3.8	− 0.6 ± 3.9	− 2.1 ± 5.1	− 0.2 ± 5.3	− 1.2 ± 6.1	0.663
		mPTT	2.4 ± 1.2	− 0.9 ± 1.4	3.7 ± 2.1	0.7 ± 2.4	11.8 ± 4.9	< 0.001^6^
	Lateral	AD-LM	− 2.1 ± 3.8	0.7 ± 2.8	− 3.8 ± 3.0	− 0.1 ± 3.5	− 7.2 ± 2.7	< 0.001^7^
		PD-LM	− 4.5 ± 2.8	0.0 ± 1.5	− 5.6 ± 2.5	1.1 ± 1.9	− 7.0 ± 3.4	< 0.001^8^
		API-LM	0.9 ± 3.8	− 0.1 ± 1.5	0.9 ± 3.0	0.2 ± 2.0	0.1 ± 2.6	0.648
		lPTT	6.6 ± 1.6	− 2.4 ± 2.0	7.7 ± 2.3	− 2.0 ± 4.0	12.7 ± 2.3	< 0.001^9^
Reader 2	Medial	AD-MM	− 0.5 ± 2.6	1.5 ± 2.7	− 0.8 ± 2.6	1.3 ± 5.1	− 4.3 ± 2.9	< 0.001^10^
		PD-MM	− 1.5 ± 3.3	1.2 ± 2.2	− 0.8 ± 3.4	0.0 ± 4.4	− 5.5 ± 5.7	< 0.001^11^
		API-MM	− 1.0 ± 4.2	− 0.5 ± 3.6	− 1.9 ± 5.1	0.1 ± 5.2	− 0.7 ± 5.6	0.730
		mPTT	2.7 ± 1.2	− 1.0 ± 1.4	4.1 ± 2.1	0.5 ± 2.2	12.3 ± 4.5	< 0.001^12^
	Lateral	AD-LM	− 2.0 ± 3.8	0.6 ± 3.6	− 4.0 ± 2.9	− 0.4 ± 3.9	− 7.1 ± 3.4	< 0.001^13^
		PD-LM	− 4.3 ± 2.8	− 0.1 ± 2.2	− 5.5 ± 2.2	1.5 ± 1.8	− 7.0 ± 3.2	< 0.001^14^
		API-LM	1.5 ± 3.7	0.2 ± 2.0	1.0 ± 2.5	0.2 ± 2.3	0.4 ± 2.9	0.514
		lPTT	6.5 ± 1.7	− 2.2 ± 2.2	7.9 ± 2.3	− 1.7 ± 3.9	12.9 ± 2.3	< 0.001^15^

2D manual reference measurements as secondary signs of PCL injury were assessed by two readers and as a function of PCL-condition PCL-condition (PCL_intact_—intact PCL; PCL_partial_—after partial PCL transection; PCL_complete_—after complete PCL transection) and loading configuration (unloaded [δ_0_] and loaded [δ_1_]). Absolute values were related to δ_0_ [PCL_intact_] to determine absolute changes (Δ_x_) for each PCL condition (Δ_x_[PCL-condition] = δ_x_[PCL-condition] − δ_0_[PCL_intact_]). Data are presented as means ± standard deviation. Statistical analysis was based on repeated measures ANOVA tests and significant findings are indicated in bold type. Sequential numbers in square brackets indicate the corresponding post-hoc details as given in Supplementary Table 2. For detailed explanations of the 2D manual reference measures, please refer to Supplementary Fig. 2. Abbreviations: PCL—posterior cruciate ligament. PTT—posterior tibial translation. lPTT (lateral PTT, i.e., PTT of the lateral femorotibial compartment). mPTT (medial PTT, i.e., PTT of the medial femorotibial compartment). AD-MM—anterior displacement of the medial meniscus. AD-LM—anterior displacement of the lateral meniscus. PD-MM—posterior displacement of the medial meniscus. PD-LM—posterior displacement of the lateral meniscus. API—anterior–posterior interval.

Significant differences as a function of PCL condition and configuration were found for the 3D Euclidean vectors vector_FT (that connects the apex of the femoral trochlea [FT] and the centre of the tibial tuberosity [TT]) and vector_ASI (that connects the femoral [fASI] and tibial axis-surface-intersections [tASI]) as well as their projections on the x-, y-, and z-axis as detailed below (see **Image Post-Processing to Quantify Joint Laxity**). Overall, significant differences were found primarily along the y-axis (anteroposterior dimension: y_ASI and y_FT [both *p* ≤ 0.001]), and along the x-axis (mediolateral dimension: x_FT [*p* = 0.008]). In the following, findings of statistical significance refer to the post-hoc comparisons to the PCL_intact_ condition.

For the PCL_intact_ condition, loading induced moderate changes in the joint with the largest and significant increases along the y-axis: Δ_1_[y_ASI] = 7.9 ± 3.2 mm; Δ_1_[y_FT] = 6.6 ± 2.4 mm. Corresponding vector lengths decreased under loading, yet non-significantly (Δ_1_[vector_ASI] = − 4.2 ± 5.2 mm [*p* = 0.415]; Δ_1_[vector_FT] = − 3.4 ± 2.8 mm [*p* = 0.101]).

In partially PCL-deficient joints, loading-induced changes in the joint were ambiguous and variable for vector lengths and their projections on the x-, y-, and z-axes. Along the y-axis, increases were moderate, yet not significant for y_ASI (Δ_1_[y_ASI] = 9.8 ± 5.8 mm [ns]) or y_FT (Δ_1_[y_FT] = 4.4 ± 5.7 mm [ns]).

In completely PCL-deficient joints, loading induced even larger and partially significant changes in the joint, with the largest increases found along the y-axis (Δ_1_[y_ASI] = 15.2 ± 5.1 mm [***]; Δ_1_[y_FT] = 12.0 ± 5.0 mm [ns]). Mean vector lengths were not homogenously altered under loading. For y_ASI and y_FT, numerous significant differences were noted between the δ_0_- and δ_1_-configurations of the various PCL conditions, while for other 3D computed vector measures, no significant loading-induced differences were found (Table 1 and Supplementary Table 1).

Overall, the 2D manual reference measurements validated the 3D computed vector measurements (Table 2). The largest mean increases were found for lPTT (lateral PTT) under loading, e.g., reader 1 [δ_1_,], PCL_intact_: 6.6 ± 1.6 mm; PCL_partial_: 7.7 ± 2.3 mm; PCL_complete_: 12.7 ± 2.3 mm (*p* < 0.001). Similarly, AD-MM (i.e., anterior displacement of the medial meniscus), AD-LM (i.e., anterior displacement of the lateral meniscus), PD-MM (i.e., posterior displacement of the medial meniscus), and PD-LM (i.e., posterior displacement of the lateral meniscus) changed significantly under loading and as a function of PCL injury (*p* ≤ 0.001 each).

## Discussion

The most important finding of this study was that increasing knee joint laxity may be reliably quantified in situ and as a function of graded PCL injury using the *MRI posterior drawer test*. Not surprisingly, quantification of altered joint kinematics was particularly relevant in the anteroposterior dimension, even though concurrent loading-induced changes in all dimensions indicate the underlying complexities of functional joint imaging.

Clinical-standard MRI techniques evaluate PCL integrity and joint status on a mere morphologic and static level without assessment of ligament function and joint laxity. As morphologic features may mask PCL injuries with pertinent residual laxity and instability, the diagnostic differentiation of partial and complete PCL injuries from the intact PCL is often challenging^9,33,39^. Stress radiography assesses altered femorotibial kinematics in PCL injury, yet suffers from numerous drawbacks such as ionizing radiation, mere 2D projections, and lack of reproducibility and accuracy^16^. As the most powerful contemporary imaging technique for the morphologic evaluation of the knee joint due to its excellent soft tissue contrast, lack of ionizing radiation, and non-invasiveness, MRI is well poised to assess a joint’s position and configuration. This study demonstrated that MRI, advanced image post-processing, and standardized loading can be brought together as the *MRI posterior drawer test* and provides combined morphologic and functional assessment of the PCL and entire knee joint.

Loading was induced by control of pressure using a commercial MRI-compatible loading device previously validated^37^. In line with the manufacturer’s instructions, we defined the biomechanical framework conditions in close emulation of the analogous and well-established stress radiographic technique^17,24^. This may facilitate inter-method comparisons and imminent translation of our findings by providing equally efficient and safe framework loading conditions in terms of loading direction and amplitude. The presence of the loading device within the bore required that a body coil be used for imaging instead of a knee coil. While knee coils provide a close fit around the joint and optimized signal-to-noise ratio, the high number of channels of modern body coils compensates for its suboptimal geometry, balances image speed and quality, and may thus fit in well with diagnostic workflows. Yet, systematic comparison of diagnostic quality of 20° of flexion in the knee coil vs. 90° of flexion in the body coil remains to be performed.

Effectual load application became evident by the loading-induced changes in the 3D computed vector and the 2D manual reference measurements.

In **PCL-intact** joints, vector projections in the anteroposterior dimension, i.e., along the y-axis, increased under loading by 6.6–7.9 mm. Manual reference measurement of lPTT by both readers revealed similar translation of approximately 6.5 mm and overall, these changes indicate the PCL’s physiological laxity. Reviewing numerous cadaveric specimen-based biomechanical studies using posterior drawer testing, Kowalczuk et al. reported mean PTTs of 5.4 mm in the PCL-intact joint. Notably, clinical studies suggest lower PTTs in intact joints, e.g. 1.3 ± 1.9 mm^36^. This discrepancy is not surprising as cadaveric specimens are only passively restrained and lack the active tone of musculotendinous structures altogether^19^.

In **partially PCL-deficient** joints, vector projections in the anteroposterior dimension were variable and ranged from 4.4 to 9.8 mm. Manual measurements of lPTT increased only slightly to a mean of 7.7–7.9 mm which is largely in agreement with the sparse literature^14,18^. Assessing PTT after bundle-wise sectioning and under a posterior force of 134 N, Kennedy et al. reported that transections of either of the PCL’s posteromedial and anterolateral bundles resulted in significant, yet only slight PTT increases of 0.9 ± 0.6 mm and 2.6 ± 1.8 mm, respectively^18^. Assessing the contributions of the individual PCL fiber regions that control PTT under loading at 90° of flexion, Covey et al. found that sectioning of the anterior and central fiber regions, while leaving the posterior fiber regions intact, only marginally increased PTT by 0.5 ± 0.4 mm and 0.2 ± 0.0 mm, respectively. Yet, once the posterior fiber regions were transected, PTT was increased substantially by 4.7–6.0 mm^7^. In our study, arthroscopic partial PCL injury was induced by transection of approximately 50% of the PCL fibers from anterior, i.e., through the anteromedial and anterolateral portals. Even though partial PCL injury had to rely on the surgeon’s feel, orientational arthroscopic and MRI measurements confirmed the partial PCL to approximately half of the PCL’s total diameter. As we did not separate(ly transect) the anterolateral or posteromedial PCL bundles, our setup is comparable to the deficiency of the PCL’s anterior and central fiber regions and may explain the only slight and variable increases in PTT. Rather than differentiating the individual contributions of the PCL fibers in biomechanical contexts, we aimed to induce standardized partial PCL injuries as defined by the extent of ligament damage. Yet, despite our best efforts to fully visualize the PCL following synovectomy, standardizing the extent of graded PCL injury proved challenging and transection may have favoured the anterolateral over the posteromedial bundle. The nature of the transection-induced partial PCL injury also limits clinical transferability because actual PCL injury necessarily involves elements of over-stretching and consecutive macro- and microstructural damage of both bundles. This could not be emulated in our experimental setup and would require more refined techniques of joint traumatization. Additionally, subsequent PTT (as determined by partial PCL injury) is not only affected by the number of injured fibers but also by the functionality of the remaining fibers, the bundles affected, the location of ligament injury, and concomitant joint injuries^9,18^. Therefore, partial PCL injuries are inherently variable and, consequently, not uniform in their imaging and clinical manifestations.

Taken together, the small and variable changes in femorotibial kinematics may be the principal reason for the failure of the *MRI posterior drawer test* to achieve the diagnostic differentiation of partial PCL injury versus other PCL conditions.

In **completely PCL-deficient** joints, vector projections in the anteroposterior dimension were significantly larger and ranged from 12.0–15.2 mm. Corresponding manual measures of PTT indicated a similar range of 11.8–12.9 mm (for the PTT of the medial and lateral compartment). At 90° of flexion, mean PTT increases of 11.7 ± 4.0 mm (under 134 N)^18^ and 12.7 ± 1.0 mm (under 200 N)^38^ have been reported before, thereby corroborating our findings. While PTT is known to increase substantially in complete PCL injury, its exact amount varies considerably and is affected by study design and methodology, reference measurements, and biomechanical framework conditions. These ill-controlled variables result in the largely variable PTT values of 7.2–18.7 mm reported in earlier studies^7–9,38^.

The **medial and lateral femorotibial compartments** behaved differently under loading. In intact and partially PCL-deficient joints, the amount of PTT was substantially higher laterally than medially, i.e., lPTT > mPTT, which may be attributed to the PCL’s role in stabilizing the joint against excessive rotation at higher flexion^11,27^. At 90° of flexion, the PCL primarily restrains internal rotation and only secondarily limits external rotation^18^, which explains larger motional translation laterally. In completely PCL-deficient joints, however, PTT was largely similar medially and laterally. Most likely, this finding is due to excessive internal rotation secondary to complete PCL injury which brings about more substantial translation of the medial compartment^18,29^. Consequently, both the mPTT and the lPTT should be determined in manual 2D reference measurements to differentiate partial from complete PCL injury. Beyond each compartment’s translation, joint rotation and flexion should be determined, too, to improve measurement validity and diagnostic differentiation of isolated and combined ligament injuries e.g., in efforts to differentiate the isolated PCL injury from the combined PCL and posterolateral corner injuries.

In this study, **advanced image post-processing techniques** were implemented to parameterize and quantify loading-induced joint changes. Beyond evaluation of the anteroposterior dimension, motional joint changes in the mediolateral and craniocaudal dimensions were assessed, too. Since joints were not constrained under loading, adaptive flexion and rotation was possible which may not be representative of the more confined whole-extremity configuration in patients. Consequently, significant changes in x_FT suggest altered mediolateral alignment and should be considered against this study’s experimental design. Nonetheless, such refined techniques provide the basis for enhanced control of otherwise ill-controlled factors such as joint position and rotation that are known to affect PTT quantification^12,18^. Thereby, validity and reproducibility of laxity measurements may be prospectively improved.

Numerous **limitations** should be recognized that potentially limit clinical applicability and may require further exploration. First, this was an *in-situ* study with obviously limited translatability to the *in-vivo* setting as active stabilizers of the joint were absent and only relatively few specimens (n = 11) had been included. Second, the manual 2D or computed 3D measurements were not validated against standard clinical or research measurements, e.g., manual or instrumented laxity measurements or stress radiographic measurements. In our specimens, side-to-side differences were not assessable either. Third, the aged cadaveric specimens might not be representative of the substantially younger clinical population with stronger PCLs. Fourth, our arthroscopic model of PCL injury bears only limited resemblance to the actual PCL injury as detailed above. Similarly, PCL injury often occurs alongside other structural joint, thus manifesting as multi-ligament injury. For example, additional posteromedial and posterolateral corner injuries substantially increase PTT^15^ and may challenge correct differentiation of intact, partial or complete PCL injuries^25^. Fifth, our post-processing technique to quantify joint kinematics only provides global estimates of PTT and does not yet allow comprehensive compartmental or regional assessment. In contrast, the 2D manual measures are well-validated imaging measures and have been applied in clinical studies of PCL function that allow assessment of compartmental motional changes^8,9^. For full exploitation of the diagnostic potential of stress MRI, the 3D joint models should be further improved to allow assessment of compartmental translation, joint rotation, and joint flexion. Once additional scientific and clinical data on the loading-induced changes of the joint have been compiled as “(in)stability patterns”, these data may be prospectively used to differentiate partial from complete injury as well as isolated from combined injury. Sixth, exact segmentations of the femur and tibia are needed, which, if performed manually, are labour-intensive and time-consuming. For prospective clinical implementation, automated segmentation approaches as suggested previously^35^ are necessary. Seventh, the clinical potential of the MRI posterior drawer test is yet unclear and requires further corroboration in clinical studies. The clinically oriented imaging framework (in terms of the clinical 3.0 T MRI scanner, coils, and MRI sequences) and the safe and efficient loading of the joint (by means of the MRI-compatible loading device) provide a solid foundation for the future clinical translation of stress MRI techniques. The next objective is to confirm our findings of physiological laxity in healthy volunteers and to assess aspects of comfort, device handleability and safety, joint fixation, and stabilization as well as measurement validity and reproducibility with the device in clinical operation. Subsequently, the diagnostic potential needs to be assessed in patients (with isolated and combined ligament injuries) and, if possible, in reference to arthroscopy as the reference standard. In these *in-vivo* studies, the clinical value of the 3D computed vector measures requires additional scientific corroboration.

In conclusion, the *MRI posterior drawer test* brings together MRI and mechanical loading, and -if complemented by additional advanced image post-processing- it allows for simultaneous assessment of ligament structure and function as well as joint laxity. Consequently, this study provides baseline normative multidimensional imaging markers of knee joint laxity as a function of PCL condition and sets the stage for subsequent *in-vivo* studies. Beyond the differentiation of acute PCL injury based on femorotibial kinematics, such functional approaches may have a prospective role in therapeutic decision-making and treatment monitoring.

## Methods

### Study design

This study was designed as an intra-individual comparative *in-situ* imaging study using knee joint specimens from body donors who had given their written informed consent and had deceased due to conditions unrelated to knee health. Approval by the local institutional review board (Ethical Committee of the Medical Faculty of Heinrich-Heine-University, Düsseldorf, Germany, 2019–682) was obtained before the study. The study has been conducted in accordance with all relevant ethical regulations and local guidelines.

### Human knee joint specimens

Macroscopically intact unfixed and unpaired human knee joint specimens (n = 11; left: 6; right: 5; female: 8; male: 3; mean age: 80 ± 8 years, range: 64–91 years) were provided by the local Institute of Anatomy (Heinrich-Heine-University Düsseldorf) and included in this study. Details of the donors’ history in terms of knee joint injury or surgery was not available. Minimum specimen number was estimated as ten following power analyses on the initial four knee joint specimens^2^. Based on the power of 0.8 and the probability of type-I-error of 0.05, the effect size (defined as the mean paired difference divided by the expected standard deviation) was determined as 1.4 after manually measuring mPTT in the unloaded and loaded configurations of the intact specimens (two-tailed procedure; online software: www.statstodo.com). Assuming larger effect sizes with increasing PCL injury and central or lateral measurements, we decided to include more than the minimum specimen number and, thus, eleven specimens.

### Loading device

**Figure 3:**
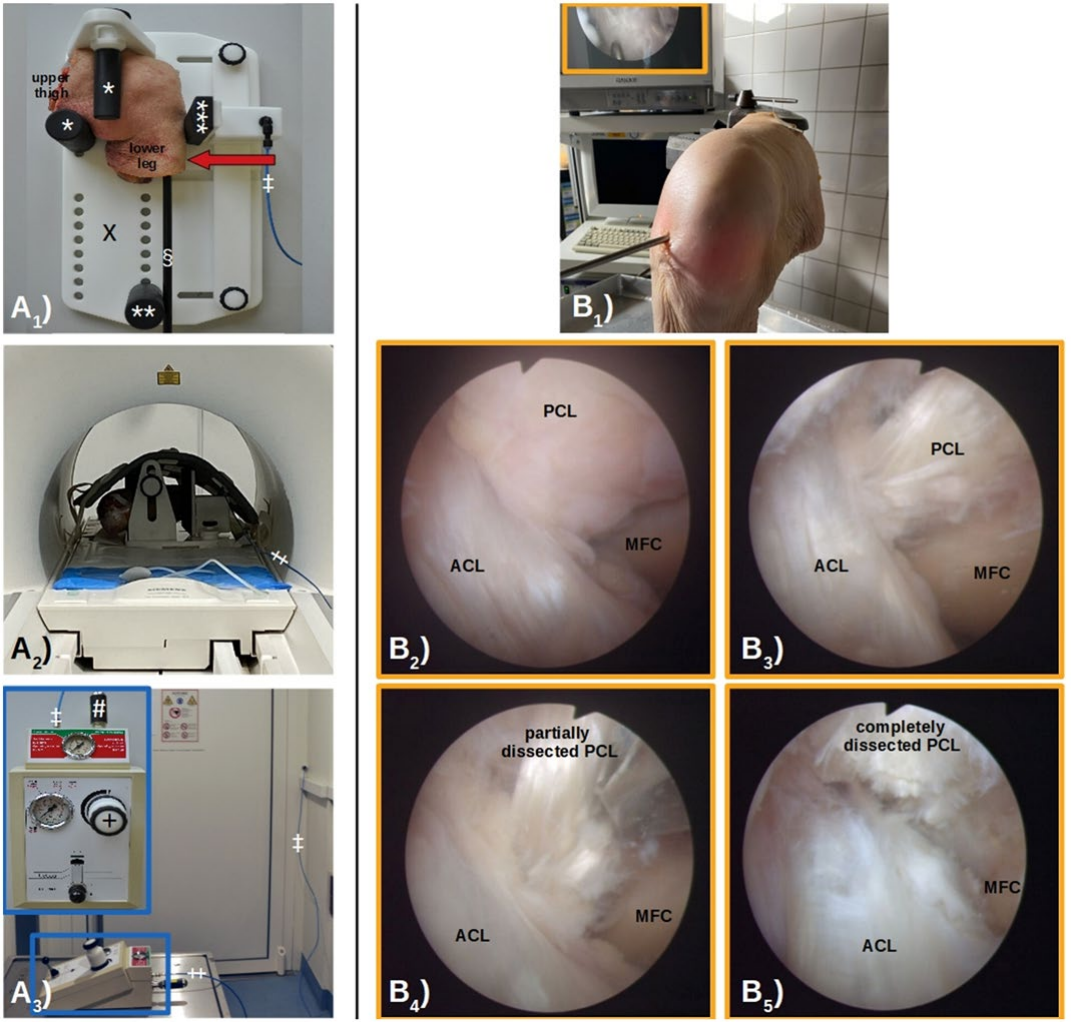
The *MRI Posterior Drawer Test*—Details of the MRI (**A**) and Arthroscopic Setup (**B**). (**A**_**1**_) Loading unit: The knee joint specimen was positioned on the base plate (X) in the lateral position at 90° of flexion and fixed with two counter-bearings at the distal upper thigh (*) and one counter-bearing at the distal lower thigh (**). Posterior loading of the tibia relative to the fixed femur (red block arrow) was induced via the padded pressure applicator (***) placed on the level of the tibial tuberosity. To compensate for the lacking distal lower thigh, a polyvinyl-chloride rod was inserted into the medullary cavity (§) to extend the tibia. The loading unit was connected to the control unit by standard pressure lines (‡). (**A**_**2**_) Fully operational setup: The specimen-loaded device was positioned centrally in the clinical MRI scanner’s bore and covered with an 18-channel body coil used for imaging. A pressure line connected the loading and control units (‡). (**A**_**3**_) Control unit: Outside of the scanner room, the control unit was connected to the loading device (‡). Once pressurized by carbon dioxide cartridges (#), pressure level (and resultant force on the joint) were controlled by a manometer ( +). Left upper box (framed in blue) indicates close-up view of the control unit. (**B**_**1**_) Arthroscopy setting: Following injection of fluid, the knee joint was accessed via the anterolateral portal. Arthroscopy tower in the background. (**B**_**2–5**_) Arthroscopic views of a right knee joint undergoing serial transections of the posterior cruciate ligament (PCL) in two separate arthroscopy sessions. Intact condition showing the PCL, the anterior cruciate ligament (ACL), and the medial femoral condyle (MFC) (**B**_**2**_). Following partial synovectomy of the PCL (**B**_**3**_), the PCL was partially transected during the first arthroscopy session by cutting approximately 50% of its cross-sectional area using arthroscopic scissors (**B**_**4**_). The functionality of the remaining PCL fibers was probed (not shown). During the second arthroscopy session, the PCL was completely transected (**B**_**5**_).

In line with the manufacturer’s instructions, a validated MRI-compatible pressure-controlled loading device was used (Stress Device SE-MR, Telos GmbH, Wölfersheim, Germany)^37^. Mechanistically, the tibia was displaced posteriorly relative to the fixed femur to emulate the *MRI posterior drawer test* (Fig. 3A1). Fixed with a padded counter-bearing on the distal medial upper thigh (aligned parallel to the tibia), the specimens were placed on the baseplate and in the lateral position at 90° of flexion. A second padded counter-bearing at the distal upper thigh (aligned parallel to the joint line) helped maintain joint flexion and stabilize the knee joint during loading, while the padded pressure applicator was positioned at the level of the tibial tuberosity in loose contact with the joint. In vivo, a third padded counter bearing above the ankle would mechanically fix the tibia, yet, in the present *in-situ* configuration, the lacking distal lower extremity was compensated for by distal extension of the tibia with a tapered polyvinyl-chloride medullary rod inserted into the medullary cavity and fixed with polymethyl-methacrylate (Technovit-3040, Heraeus-Kulzer, Wehrheim, Germany). Centrally positioned in the scanner’s bore (Fig. 3A2), the specimen-loaded device was connected to the control unit located outside of the scanner room (Fig. 3A3). Once the device was pressurized by connecting standard carbon dioxide cartridges (Telos GmbH, liquid mass of 16 g at 50.9 bar, equal to an expanded volume of 8.7 l at 15 °C) to the control unit, forces were directly transferred to the joint by posterior displacement of the padded pressure applicator.

### Image data acquisition

Imaging was performed on a clinical 3.0-T MRI scanner (Magnetom Prisma, Siemens Healthineers, Erlangen, Germany) using an 18-channel body coil (Body 18 SlideConnect, Siemens Healthineers) and a 32-channel spine coil (Spine 32 DirectConnect, Siemens Healthineers) centered on the joint and placed above and below the specimen-loaded device (Fig. 3A2).

Each specimen was subject to three MRI measurement series, i.e., intact (PCL_intact_), partially transected (PCL_partial_), and completely transected (PCL_complete_), in two configurations each, i.e., unloaded (δ_0_) and loaded (δ_1_). After loading and prior to imaging, 5 min of equilibration were allowed as an empirical time frame that struck a sensible balance between loading-induced tissue adaptation and additional time demand. While the field of view was adapted to the specimen’s anatomy, slice geometry and sequence parameters were unchanged between the δ_0_- and δ_1_-configurations. Scout views were used to ascertain constant joint flexion and standardize image conditions for each configuration and MRI measurement series. Acquired for each configuration, the imaging protocol as outlined in Table 3 was performed in line with clinical standard routines and included Proton Density-weighted fat-saturated and T1-weighted sequences. Coronal and axial sequences were used to guide the parasagittal sections along the course of the PCL.

**Table 3:** Acquisition parameters of MR sequences.

	PDW fs	PDW fs	PDW fs	PDW fs	T1-w
Sequence type	2D TSE	2D TSE	2D TSE	2D TSE	2D SE
Orientation	Cor	ax	Sag	Parasag (*)	Sag
Repetition time (ms)	4020	4000	4020	4020	864
Echo time (ms)	43	34	43	43	13
Turbo spin-echo factor	9	9	9	9	n/a
Field of view (mm)	160 × 160	160 × 160	160 × 160	160 × 160	200 × 200
Acquisition matrix (pixels)	320 × 320	320 × 320	320 × 320	320 × 320	320 × 320
Reconstruction matrix (pixels)	320 × 320	320 × 320	320 × 320	320 × 320	320 × 320
Scan percentage (%)	100	100	100	100	100
Flip angle (°)	150	150	150	150	90/160
Number of signal averages (n)	1	1	1	1	1
Slices (n)	30	30	30	30	40
Pixel size (mm/pixel)	0.5 × 0.5	0.5 × 0.5	0.5 × 0.5	0.5 × 0.5	0.6 × 0.6
Slice thickness/Gap (mm)	3.0/0.3	3.0/0.3	3.0/0.3	3.0/0.3	3.0/0.3
Duration (min:sec)	02:13	02:13	02:13	02:13	03:47

PDW—Proton Density-weighted, fs—fat-saturated, TSE—turbospin-echo, SE—spin-echo, w—weighted, cor—coronal, ax—axial, (para)sag—(para)sagittal, n/a—not applicable.

(*) aligned to the course of the PCL.

In the intact δ_0_-configuration, a clinical radiologist (SN, eight years of experience in musculoskeletal imaging) confirmed gross integrity of the pertinent intra- and periarticular knee joint structures and evaluated the presence of any signs of pre-existent PCL injury, i.e., aberrant PCL configuration, diameter, or course, focal fiber discontinuity, or excessive PTT^21,41^. These signs had been defined as exclusion criteria, yet were absent in all specimens. Following completion of the imaging protocol at the δ_0_-configuration, the padded pressure applicator was actuated to the pressure level of 2.3 bar, translating to effective posterior forces on the joint of 147 N (= 15 kP) as validated by the manufacturer.

The imaging protocol was obtained for each condition and configuration so that a total of six MRI measurement series were performed per specimen within 48 h. In-between the imaging sessions, the specimens were kept refrigerated at 4 °C and thoroughly warmed to room temperature before scanning. Per specimen, PCL condition, and loading configuration, scanning time was approximately 15 min and, consequently, total magnet time for each specimen was approximately 90 min.

### Arthroscopic model of graded PCL injury

Arthroscopic preparation and sequential transection of the PCL was performed by LMW (orthopaedic surgeon with 5 years of experience in arthroscopy) (Fig. 3B1). After establishing access to the joint via the anterolateral and anteromedial portals, the PCL was identified (Fig. 3B2) and synovectomized using curved-tip punch forceps (Arthrex, Naples, FL, US) for optimized visualization prior to transection (Fig. 3B3). To assess pre-existent laxity, the PCL was probed prior to transection. For partial PCL transection, straight-tip arthroscopic scissors (Arthrex) were used to carefully transect approximately 50% of the ligament’s diameter at mid substance just cranial to the cruciate ligament intersection, i.e., at mid-substance, and orientational arthroscopic and imaging measurements confirmed the partial PCL injury. Of note, the two individual PCL bundles were not identified or separated during transection (Fig. 3B4). Following partial transection, the remaining PCL fibers were probed to ascertain functional integrity. For complete PCL transection, the remaining PCL fibers were similarly transected (Fig. 3B5). Following each arthroscopy session, excess fluid was removed and both portals were sutured.

### Image post-processing to quantify joint laxity

To assess and quantify joint changes as a function of loading, specimen-specific 3D joint models were implemented using Python software (v3.7.3, Python Software Foundation, Wilmington, Del, US). DK (medical student, 1 year of experience in musculoskeletal imaging) performed the segmentations using the brush tool and polygon mode of ITK-SNAP software (version 3.8.0, Cognitica, Philadelphia, PA, US)^42^. Femoral and tibial bone outlines were manually segmented on sagittal T1-weighted sequences for each specimen, configuration (i.e., δ_0_ and δ_1_), and condition (i.e., PCL_intact_, PCL_partial_, and PCL_complete_). Based on the outlines of femur and tibia, the coordinates of the following anatomic landmarks were manually registered in Cartesian coordinate systems: (1) most proximal extension of the femoral trochlea (i.e., its tip) that was still covered by articular cartilage (FT), (2) centre of the tibial tuberosity (TT), (3) centers of proximal and distal femur to define the central femoral bone axis, and (4) centers of proximal and distal tibia to define the central tibial bone axis. For (3) and (4), coordinates were selected at the height of the meta-diaphyseal junction and at the most distant diaphyseal extension that was still visualized in the field-of-view. Segmentation outlines and registered coordinates were reviewed for accuracy and, if necessary, corrected, by LMW. To assess intra-reader reproducibility, registration of these coordinates was repeated in a blinded manner on all 11 specimens after 12 weeks. Except for the centre of the TT (2.19 ± 2.02 mm), inter-measurement deviations of coordinates were low and averaged between 0.42 and 0.69 mm. Please see Supplementary Table 4for additional details.

Additionally, two fixpoints were automatically computed as the intersections of the central femoral and tibial bone axes and the corresponding articular surfaces of the femoral and tibial cortices, i.e., the femoral and tibial axis-surface-intersections (fASI, tASI).

To quantify loading-induced joint changes in the three dimensions, i.e., anteroposterior (yz-plane), mediolateral (xz-plane), and craniocaudal (xy-plane), 3D Euclidean vectors were calculated between FT and TT, i.e., between (i) and (ii) (vector_FT), and between fASI and tASI (vector_ASI). Additionally, each vectors’ magnitude and projections onto the Cartesian x-, y-, and z-axes were determined, too. Based on voxel size (0.6 × 0.6 × 3.0 mm^3^) and inter-slice gap (0.3 mm), voxel-wise measures were converted to millimetres. Exemplary segmentation outlines, anatomic landmarks, computed fixpoints, vectors, and vector projections are visualized in Supplementary Fig. 1. A more detailed description of the post-processing methodology is attached in the Supplementary Material as Supplementary Text 1 (Detailed Description of Post-Processing and Image Analysis.

### Manual reference measurements to quantify joint laxity

For each specimen, configuration, and condition, secondary signs of PCL injury were quantified by means of manual 2D reference measurements obtained by LMW and DK. These signs indicate altered displacement of the tibia versus the femur^9,26^ and were measured on the central medial and lateral slices of the respective femorotibial compartments^8,9^ using the manual image analysis toolbox of the in-house picture archiving and communications system (Sectra Workstation101, IDS7, Linköping, Sweden). The slices on which the measurements were performed were the same for each set of measurements and the measurements were defined as follows:

(1) Anterior and posterior displacements of the lateral **(AD-LM, PD-LM)** and medial meniscus **(AD-MM, PD-MM)** indicate the horizontal distances between the anterior or posterior borders of the lateral or medial tibial plateau and the base of the respective anterior or posterior meniscal horns. (2) Anterior–posterior intervals **(API)** of the lateral and medial meniscus **(API-LM, API-MM)** indicate the horizontal distances between the inner edges of the anterior and posterior horns of the lateral or medial meniscus. (3) Lateral and medial PTT **(lPTT, mPTT)** indicate the horizontal distances between the posterior lateral or medial tibial plateau and the respective posterior femoral condyle. Supplementary Fig. 2 gives representative examples of the manual reference measures. For AD-, PD-, and API-related measures, posterior displacement relative to the tibial plateau is indicated by positive values, while for PTT-related measures, posterior tibial displacement relative to the femur is indicated by positive values. As PCL condition and configuration were easily identified on MR images, blinding of the readers was not feasible.

### Statistical analysis

Statistical analysis was performed by LMW using GraphPad Prism (v5.0, San Diego, CA, US).

Data are presented as means ± standard deviations. For each condition and configuration, absolute differences were referenced to δ_0_ [PCL_intact_]. Consequently, absolute differences (Δ_x_) were calculated as Δ_x_[PCL_condition_] = δ_x_[PCL_condition_] − δ_0_[PCL_intact_]. Assuming normal distributions of the 3D computed vector and 2D manual reference measures, quantitative measures for each configuration and condition were assessed specimen-wise, using repeated measures ANOVA (two-sided) with Bonferroni’s test for pairwise post-hoc comparisons. The level of significance was set to *p* ≤ 0.01 to decrease the number of statistically significant, yet clinically irrelevant findings, and further stratified as 0.01 ≥ *p* > 0.001 (**) and *p* ≤ 0.001 (***).

## Supplementary Information

Supplementary Information.

## Data Availability

The main data supporting the results in this study are available within the paper and its Supplementary Information. Any additional datasets generated and analyzed in this study are available from the corresponding author on reasonable request.

## Code availability

The software routines used for computational image analysis and graphical output are available for research purposes from the corresponding author upon reasonable request.

## References

[1] Akisue T, Kurosaka M, Yoshiya S, Kuroda R, Mizuno K (2001). Evaluation of healing of the injured posterior cruciate ligament: Analysis of instability and magnetic resonance imaging. Arthroscopy.

[2] Allaire R, Muriuki M, Gilbertson L, Harner CD (2008). Biomechanical consequences of a tear of the posterior root of the medial meniscus Similar to total meniscectomy. J. Bone Joint Surg. Am..

[3] Amis AA, Gupte CM, Bull AMJ, Edwards A (2006). Anatomy of the posterior cruciate ligament and the meniscofemoral ligaments. Knee Surg. Sports Traumatol. Arthrosc..

[4] Anderson DD, Chubinskaya S, Guilak F (2011). Post-traumatic osteoarthritis: improved understanding and opportunities for early intervention. J. Orthop. Res..

[5] Bedi A, Musahl V, Cowan JB (2016). Management of posterior cruciate ligament injuries: An evidence-based review. J. Am. Acad. Orthop. Surg..

[6] Blalock D, Miller A, Tilley M, Wang J (2015). Joint instability and osteoarthritis. Clin. Med. Insights Arthritis Musculoskelet Disord..

[7] Covey DC, Sapega AA, Riffenburgh RH (2008). The effects of sequential sectioning of defined posterior cruciate ligament fiber regions on translational knee motion. Am. J. Sports Med..

[8] Degnan AJ, Maldjian C, Adam RJ, Harner CD (2014). Passive posterior tibial subluxation on routine knee MRI as a secondary sign of PCL tear. Radiol. Res. Practice..

[9] DePhillipo NN, Cinque ME, Godin JA, Moatshe G, Chahla J, LaPrade RF (2018). Posterior tibial translation measurements on magnetic resonance imaging improve diagnostic sensitivity for chronic posterior cruciate ligament injuries and graft tears. Am. J. Sports Med..

[10] Esmaili Jah AA, Keyhani S, Zarei R, Moghaddam AK (2005). Accuracy of MRI in comparison with clinical and arthroscopic findings in ligamentous and meniscal injuries of the knee. Acta Orthop. Belg..

[11] Fukagawa S, Matsuda S, Tashiro Y, Hashizume M, Iwamoto Y (2010). Posterior displacement of the tibia increases in deep flexion of the knee. Clin. Orthop. Relat. Res..

[12] Grassmayr MJ, Parker DA, Coolican MRJ, Vanwanseele B (2008). Posterior cruciate ligament deficiency: Biomechanical and biological consequences and the outcomes of conservative treatment. A systematic review. J. Sci. Med. Sport..

[13] Gross ML, Grover JS, Bassett LW, Seeger LL, Finerman GA (1992). Magnetic resonance imaging of the posterior cruciate ligament. Clinical use to improve diagnostic accuracy. Am. J. Sports Med..

[14] Hewett TE, Noyes FR, Lee MD (1997). Diagnosis of complete and partial posterior cruciate ligament ruptures. Stress radiography compared with KT-1000 arthrometer and posterior drawer testing. Am. J. Sports Med..

[15] Jackman T, LaPrade RF, Pontinen T, Lender PA (2008). Intraobserver and interobserver reliability of the kneeling technique of stress radiography for the evaluation of posterior knee laxity. Am. J. Sports Med..

[16] James EW, Williams BT, LaPrade RF (2014). Stress radiography for the diagnosis of knee ligament injuries: A systematic review. Clin. Orthop. Relat Res..

[17] Jung TM, Reinhardt C, Scheffler SU, Weiler A (2006). Stress radiography to measure posterior cruciate ligament insufficiency: A comparison of five different techniques. Knee Surg. Sports Traumatol. Arthrosc..

[18] Kennedy NI, Wijdicks CA, Goldsmith MT (2013). Kinematic analysis of the posterior cruciate ligament, part 1: The individual and collective function of the anterolateral and posteromedial bundles. Am. J. Sports Med..

[19] Kowalczuk M, Leblanc M-C, Rothrauff BB (2015). Posterior tibial translation resulting from the posterior drawer manoeuver in cadaveric knee specimens: A systematic review. Knee Surg. Sports Traumatol. Arthrosc..

[20] Laoruengthana A, Jarusriwanna A (2012). Sensitivity and specificity of magnetic resonance imaging for knee injury and clinical application for the Naresuan University Hospital. J. Med. Assoc. Thai..

[21] LaPrade CM, Civitarese DM, Rasmussen MT, LaPrade RF (2015). Emerging updates on the posterior cruciate ligament: A review of the current literature. Am. J. Sports Med..

[22] Lee YS, Han SH, Jo J, Kwak K-S, Nha KW, Kim JH (2011). Comparison of 5 different methods for measuring stress radiographs to improve reproducibility during the evaluation of knee instability. Am. J. Sports Med..

[23] Logan M, Williams A, Lavelle J, Gedroyc W, Freeman M (2004). The effect of posterior cruciate ligament deficiency on knee kinematics. Am. J. Sports Med..

[24] Margheritini F, Mancini L, Mauro CS, Mariani PP (2003). Stress radiography for quantifying posterior cruciate ligament deficiency. Arthroscopy.

[25] Margheritini F, Mariani PP (2003). Diagnostic evaluation of posterior cruciate ligament injuries. Knee Surg. Sports Traumatol. Arthrosc..

[26] Masuda S, Furumatsu T, Okazaki Y (2018). Medial meniscus posterior root tear induces pathological posterior extrusion of the meniscus in the knee-flexed position: An open magnetic resonance imaging analysis. Orthop. Traumatol. Surg. Res..

[27] Noyes FR, Stowers SF, Grood ES, Cummings J, VanGinkel LA (1993). Posterior subluxations of the medial and lateral tibiofemoral compartments. An in vitro ligament sectioning study in cadaveric knees. Am. J. Sports Med..

[28] Orakzai SH, Egan CM, Eustace S, Kenny P, O’flanagan SJ, Keogh P (2010). Correlation of intra-articular osseous measurements with posterior cruciate ligament length on MRI scans. Br. J. Radiol..

[29] Pache S, Aman ZS, Kennedy M (2018). Posterior cruciate ligament: Current concepts review. Arch. Bone Jt. Surg..

[30] Parkar AP, Alcalá-Galiano A (2016). Rupture of the posterior cruciate ligament: Preoperative and postoperative assessment. Semin. Musculoskelet Radiol..

[31] Parkar AP, Vanhoenacker FM, Adriaensen ME (2013). Bilateral mucoid degeneration of the posterior cruciate ligaments. JBR-BTR..

[32] Patten RM, Richardson ML, Zink-Brody G, Rolfe BA (1994). Complete vs partial-thickness tears of the posterior cruciate ligament: MR findings. J. Comput. Assist. Tomogr..

[33] Rodriguez W, Vinson EN, Helms CA, Toth AP (2008). MRI appearance of posterior cruciate ligament tears. AJR Am. J. Roentgenol..

[34] Rubinstein RA, Shelbourne KD, McCarroll JR, VanMeter CD, Rettig AC (1994). The accuracy of the clinical examination in the setting of posterior cruciate ligament injuries. Am. J. Sports Med..

[35] Schock J, Kopaczka M, Agthe B, et al. A Method for Semantic Knee Bone and Cartilage Segmentation with Deep 3D Shape Fitting Using Data from the Osteoarthritis Initiative. In: Reuter M, Wachinger C, Lombaert H, Paniagua B, Goksel O, Rekik I, eds. *Shape in Medical Imaging*. Lecture Notes in Computer Science. Springer International Publishing; 2020:85–94. 10.1007/978-3-030-61056-2_7

[36] Schulz MS, Steenlage ES, Russe K, Strobel MJ (2007). Distribution of posterior tibial displacement in knees with posterior cruciate ligament tears. J. Bone Joint Surg. Am..

[37] Seebauer CJ, Bail HJ, Rump JC, Hamm B, Walter T, Teichgräber UKM (2013). Ankle laxity: Stress investigation under MRI control. Am. J. Roentgenol..

[38] Sekiya JK, Whiddon DR, Zehms CT, Miller MD (2008). A clinically relevant assessment of posterior cruciate ligament and posterolateral corner injuries. Evaluation of isolated and combined deficiency. J. Bone Joint Surg. Am..

[39] Servant CTJ, Ramos JP, Thomas NP (2004). The accuracy of magnetic resonance imaging in diagnosing chronic posterior cruciate ligament injury. Knee.

[40] Tewes DP, Fritts HM, Fields RD, Quick DC, Buss DD (1997). Chronically injured posterior cruciate ligament: Magnetic resonance imaging. Clin. Orthop. Relat Res..

[41] Wilson KJ, Fripp J, Lockard CA (2019). Quantitative mapping of acute and chronic PCL pathology with 3 T MRI: A prospectively enrolled patient cohort. J. Exp. Orthop..

[42] Yushkevich PA, Piven J, Hazlett HC (2006). User-guided 3D active contour segmentation of anatomical structures: Significantly improved efficiency and reliability. Neuroimage.

