# Supplementary material for: The MRI posterior drawer test to assess posterior cruciate ligament functionality and knee joint laxity
Source: Sci Rep. 2021 Oct 4;11:19687. doi: 10.1038/s41598-021-99216-w (PMC8490383; doi:10.1038/s41598-021-99216-w)
Supplement: Supplementary file 1 — Supplementary Information. [file 41598_2021_99216_MOESM1_ESM.docx]

**Supplementary Material**

**Supplementary Figures**


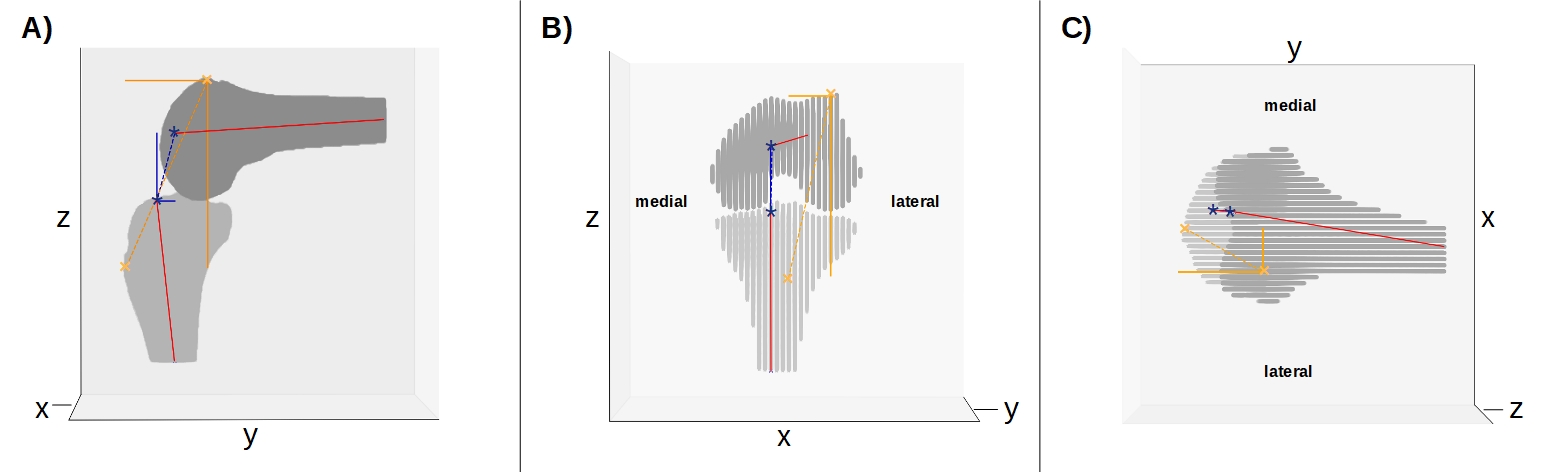


**Supplementary Figure 1: Image Post-Processing Methodology to Quantify Joint Laxity**

In a representative PCL-intact knee joint, the manually segmented bone contours of the femur (dark grey) and tibia (light grey) are displayed along the Cartesian planes in the anteroposterior (yz-plane, sagittal view [A]), mediolateral (xz-plane, coronal view [B]), and craniocaudal dimensions (xy-plane, axial view [C]). The femoral and tibial central bone axes (solid red lines) were registered and used to compute the axis-surface-intersections (ASI) of the femur (fASI) and tibia (tASI) (blue asterisks). Anatomic landmarks were identified as the tip of the cartilage-covered femoral trochlea (FT) and the centre of the tibial tuberosity (TT) (orange crosses). 3D Euclidean vectors connected FT and TT (vector n°1 = vector_FT; dashed orange lines) and fASI and tASI (vector n°2 = vector_ASI; dashed blue lines). The projections of vector_FT and vector_ASI on the x-, y-, and z-axes are indicated by the solid orange and blue lines parallel to the Cartesian axes. These projections were quantified as x_FT, x_ASI, y_FT, y_ASI, z_FT, and z_ASI. Positive values indicate a more lateral, anterior, and proximal position of the femoral (FT, fASI) to the tibial reference coordinate (TT, tASI). Axes range from 0-220 (x-axis), 0-135 (y-axis), and 0-210 (z-axis) pixels. Due to interslice gaps following image reconstruction, bone contours of femur and tibia appear sliced (B, C). Same left knee joint specimen as in **Figures 1 and 2** and **Supplementary Figure 2**.

**
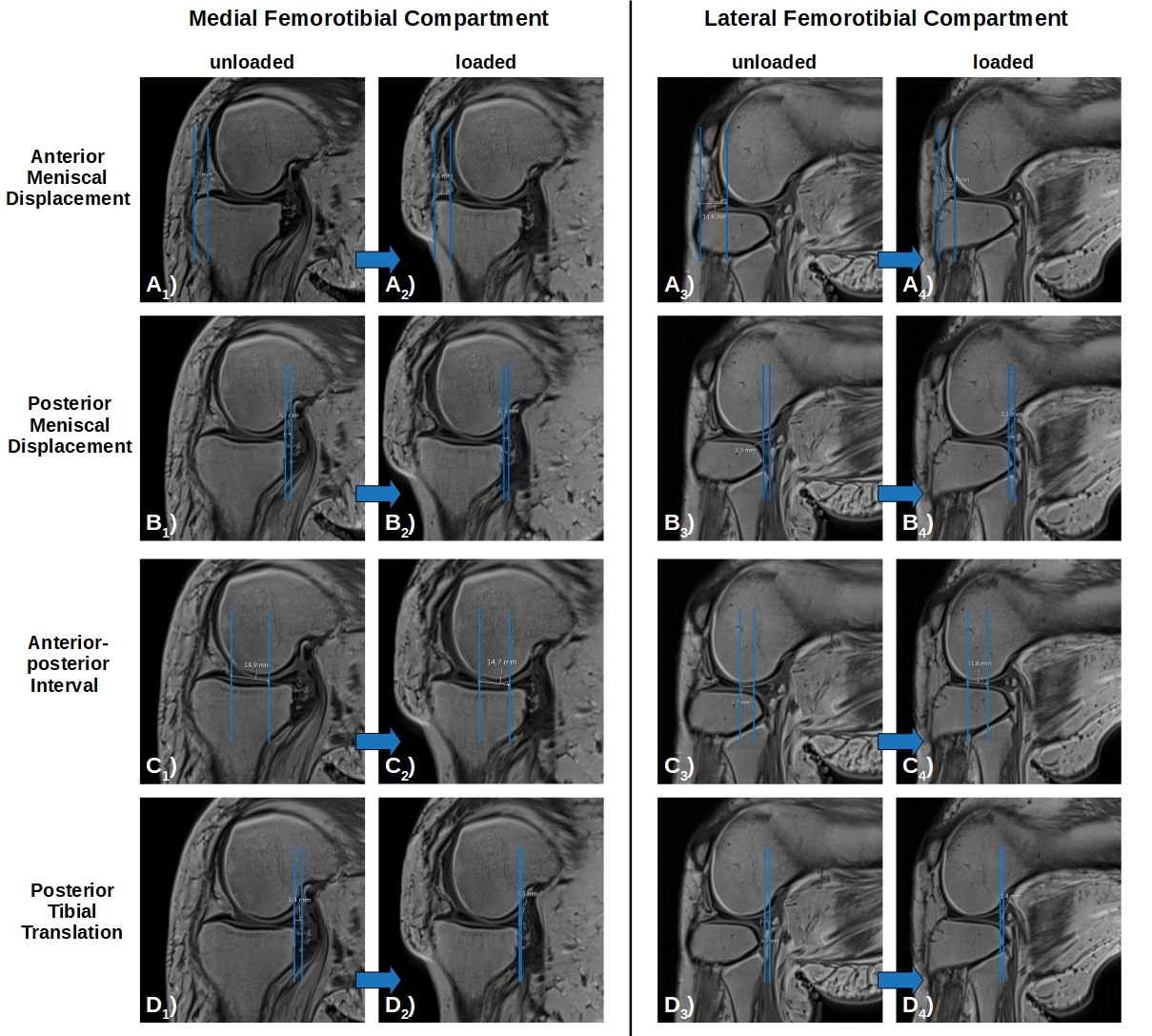
**

Supplementary Figure 2: 2D Manual Reference Measurements to Quantify Joint Laxity

Displayed are mid-medial and mid-lateral sagittal T1-weighted sequences of a PCL-intact knee joint in the unloaded (A1-D1, A3-D3) and loaded (A2-D2, A4-D4) configurations. Blue block arrows indicate the direction of force under loading. Tibial translation, i.e., changes of the tibia relative to other anatomic landmarks, was quantified relative to the anterior meniscal horns (anterior meniscal displacement of the medial meniscus [A1, A2] and the lateral meniscus [A3, A4]), the posterior meniscal horns (posterior meniscal displacement of the medial meniscus [B1, B2] and the lateral meniscus [B3, B4]), in terms of the anterior-posterior intervals of the medial meniscus (C1, C2) and the lateral meniscus (C3, C4), and relative to the medial (mPTT; D1, D2) and lateral femoral condyles (lPTT; D3, D4). Anterior and posterior meniscal displacement measures the horizontal distances between tangential lines at the base of the anterior or posterior meniscus horns and the corresponding articular surface borders of the tibial plateau. Anterior-posterior interval measures the horizontal distances between the inner edges of the anterior and posterior horns of the medial or lateral menisci. Positive or negative values indicate a more posterior or anterior displacement of the meniscal horns relative to the tibial plateau. MPTT and lPTT quantify the horizontal distances between tangential lines at the posterior borders of the tibial plateau and femoral condyles in the mid-medial or mid-lateral femorotibial compartments. Positive values indicate more posterior displacement of the tibial plateau relative to the femoral condyle. Units of measurements are mm. Same left knee joint specimen as in Figures 1 and 2 and Supplementary Figure 1. Abbreviations: PCL – posterior cruciate ligament; PTT – posterior tibial translation; lPTT (lateral PTT); mPTT (medial PTT).

**Supplementary Tables**

**Supplementary Table 1: Bonferroni's Post-Hoc Details of 3D Computed Vector Measures.**

Given are the details of Bonferroni’s multiple comparisons tests following repeated measures ANOVA as detailed in **Table 1**. The level of significance (*p*≤0.01) was further stratified as 0.01≥p>0.001 (**) and p≤0.001 (***), with significant findings indicated in **bold type**. Abbreviations: PCL – posterior cruciate ligament. Vector projections on Cartesian x-axis (i.e., anteroposterior dimension; “x_FT”), and y-axis (i.e., mediolateral dimension; “y_FT”, “y_ASI”).

| Bonferroni's post-hoc test | 3D Computed Vector Measures | | |
| --- | --- | --- | --- |
|  | **y_ASI [1]** | **x_FT [2]** | **y_FT [3]** |
| Δ_1_ PCL_intact_ vs. Δ_0_ PCL_partial_ | ****** | ns | ******* |
| Δ_1_ PCL_intact_ vs. Δ_1_ PCL_partial_ | ns | ns | ns |
| Δ_1_ PCL_intact_ vs. Δ_0_ PCL_complete_ | ******* | ns | ******* |
| Δ_1_ PCL_intact_ vs. Δ_1_ PCL_complete_ | ******* | ns | ns |
| Δ_0_ PCL_partial_ vs. Δ_1_ PCL_partial_ | ******* | ns | ns |
| Δ_0_ PCL_partial_ vs. Δ_0_ PCL_complete_ | ns | ns | ns |
| Δ_0_ PCL_partial_ vs. Δ_1_ PCL_complete_ | ******* | ns | ******* |
| Δ_1_ PCL_partial_ vs. Δ_0_ PCL_complete_ | ******* | ns | ns |
| Δ_1_ PCL_partial_ vs. Δ_1_ PCL_complete_ | ns | ns | ******* |
| Δ_0_ PCL_complete_ vs. Δ_1_ PCL_complete_ | ******* | ns | ******* |

**Supplementary Table 2: Bonferroni's Post-Hoc Details of 2D Manual Reference Measures.**

Given are the details of Bonferroni’s multiple comparisons tests following repeated measures ANOVA as detailed in **Table 2**. The level of significance (*p*≤0.01) was further stratified as 0.01≥p>0.001 (**) and p≤0.001 (***), with significant findings indicated in **bold type**. Abbreviations: PCL – posterior cruciate ligament. PTT – posterior tibial translation; lPTT (lateral PTT); mPTT (medial PTT). AD-MM – anterior displacement of the medial meniscus. AD-LM – anterior displacement of the lateral meniscus. PD-MM – posterior displacement of the medial meniscus. PD-LM – posterior displacement of the lateral meniscus. API – anterior-posterior interval.

| Bonferroni's post-hoc test | 2D Manual Reference Measures | | | | | | | | | | | |
| --- | --- | --- | --- | --- | --- | --- | --- | --- | --- | --- | --- | --- |
|  | **Reader 1** | | | | | | **Reader 2** | | | | | |
|  | **AD-MM [4]** | **PD-MM [5]** | **mPTT [6]** | **AD-LM [7]** | **PD-LM [8]** | **lPTT [9]** | **AD-MM [10]** | **PD-MM [11]** | **mPTT [12]** | **AD-LM [13]** | **PD-LM [14]** | **lPTT [15]** |
| Δ_1_ PCL_intact_ vs.  Δ_0_ PCL_partial_ | ns | ns | ****** | ns | ******* | ******* | ns | ns | ****** | ns | ******* | ******* |
| Δ_1_ PCL_intact_ vs.  Δ_1_ PCL_partial_ | ns | ns | ns | ns | ns | ns | ns | ns | ns | ns | ns | ns |
| Δ_1_ PCL_intact_ vs.  Δ_0_ PCL_complete_ | ns | ns | ns | ns | ******* | ******* | ns | ns | ns | ns | ******* | ******* |
| Δ_1_ PCL_intact_ vs.  Δ_1_ PCL_complete_ | ****** | ns | ******* | ******* | ns | ******* | ns | ns | ******* | ******* | ns | ******* |
| Δ_0_ PCL_partial_ vs.  Δ_1_ PCL_partial_ | ns | ns | ******* | ****** | ******* | ******* | ns | ns | ******* | ****** | ******* | ******* |
| Δ_0_ PCL_partial_ vs.  Δ_0_ PCL_complete_ | ns | ns | ns | ns | ns | ns | ns | ns | ns | ns | ns | ns |
| Δ_0_ PCL_partial_ vs.  Δ_1_ PCL_complete_ | ******* | ******* | ******* | ******* | ******* | ******* | ******* | ******* | ******* | ******* | ******* | ******* |
| Δ_1_ PCL_partial_ vs.  Δ_0_ PCL_complete_ | ns | ns | ns | ns | ******* | ******* | ns | ns | ****** | ns | ******* | ******* |
| Δ_1_ PCL_partial_ vs.  Δ_1_ PCL_complete_ | ****** | ns | ******* | ns | ns | ******* | ns | ns | ******* | ns | ns | ******* |
| Δ_0_ PCL_complete_ vs.  Δ_1_ PCL_complete_ | ******* | ****** | ******* | ******* | ******* | ******* | ******* | ****** | ******* | ******* | ******* | ******* |

**Supplementary Table 3: Absolute Values of 3D Computed Vector and 2D Manual Reference Measures as a Function of PCL Injury and Loading.**

Absolute values of 3D computed vector measures and 2D manual reference measures are given as a function of PCL-condition, i.e., PCL_intact_, PCL_partial_, and PCL_complete_, and configuration, i.e., unloaded [δ_0_] and loaded [δ_1_]. Statistical analysis was based on repeated measures ANOVA of the absolute values as a function of PCL-condition and configuration. Data are presented as means ± standard deviation. Significant findings are indicated in **bold type**. Abbreviations: PCL – posterior cruciate ligament. Vector_ASI – vector between the femoral and the tibial axis-surface-intersection. Vector_FT – vector between the apex of the femoral trochlea and the centre of the tibial tuberosity. Vector projections on Cartesian x-axis (i.e., anteroposterior dimension; “x_FT”, “x_ASI”), y-axis (i.e., mediolateral dimension; “y_FT”, “y_ASI”), and z-axis (i.e., craniocaudal dimension; “z_FT”, “z_ASI”). PTT – posterior tibial translation; lPTT (lateral PTT); mPTT (medial PTT). AD-MM – anterior displacement of the medial meniscus. AD-LM – anterior displacement of the lateral meniscus. PD-MM – posterior displacement of the medial meniscus. PD-LM – posterior displacement of the lateral meniscus. API – anterior-posterior interval.

|  | | Measure [mm] | PCL_intact_ | | PCL_partial_ | | PCL_complete_ | | p-value |
| --- | --- | --- | --- | --- | --- | --- | --- | --- | --- |
|  |  |  | **δ_0_** | **δ_1_** | **δ_0_** | **δ_1_** | **δ_0_** | **δ_1_** |  |
| 3D Computed Vector Measures | **Axis-Surface-Intersections** | **vector_ASI** | 44.4 ± 3.4 | 40.2 ± 5.3 | 42.4 ± 3.1 | 42.3 ± 5.2 | 42.7 ± 3.1 | 41.8 ± 3.3 | 0.092 |
|  |  | **x_ASI** | -1.1 ± 6.0 | -2.8 ± 6.6 | -0.8 ± 5.0 | 0.02 ± 5.9 | 3.3 ± 5.4 | -1.7 ± 7.6 | 0.791 |
|  |  | **y_ASI** | -10.4 ± 4.9 | -2.5 ± 4.7 | -8.7 ± 4.9 | -0.7 ± 5.3 | -10.0 ± 4.6 | 4.7 ± 6.3 | **< 0.001** |
|  |  | **z_ASI** | 42.5 ± 3.4 | 39.2 ± 5.3 | 40.9 ± 2.9 | 41.7 ± 5.0 | 40.8 ± 3.1 | 40.4 ± 2.8 | 0.178 |
|  | **Anatomic Landmarks** | **vector_FT** | 111.8 ± 5.4 | 108.4 ± 3.9 | 112.0 ± 7.1 | 110.5 ± 12.4 | 114.2 ± 4.6 | 106.9 ± 3.7 | 0.087 |
|  |  | **x_FT** | 13.8 ± 8.1 | 8.4 ± 5.4 | 15.3 ± 6.8 | 11.7 ± 4.8 | 15.9 ± 9.2 | 10.2 ± 10.8 | **0.004** |
|  |  | **y_FT** | -48.3 ± 4.3 | -41.7 ± 5.1 | -49.5 ± 5.9 | -43.9 ± 7.3 | -49.6 ± 4.6 | -36.3 ± 5.3 | **< 0.001** |
|  |  | **z_FT** | 99.5 ± 5.2 | 99.5 ± 3.3 | 99.0 ± 5.8 | 100.6 ± 10.8 | 101.2 ± 3.8 | 99.4 ± 2.8 | 0.929 |
| 2D Manual Reference Measures | **Reader 1** | **AD-MM** | 6.2 ± 2.2 | 5.6 ± 2.1 | 7.8 ± 3.5 | 5.7 ± 3.1 | 7.5 ± 4.9 | 1.5 ± 2.4 | **< 0.001** |
|  |  | **PD-MM** | 2.2 ± 2.5 | 1.1 ± 3.8 | 3.2 ± 1.0 | 1.7 ± 2.6 | 2.1 ± 2.6 | -3.3 ± 5.7 | **< 0.001** |
|  |  | **API-MM** | 20.3 ± 5.7 | 19.4 ± 5.3 | 19.7 ± 4.8 | 18.3 ± 5.6 | 20.2 ± 5.3 | 19.1 ± 4.4 | 0.684 |
|  |  | **mPTT** | -4.1 ± 2.8 | -1.8 ± 3.2 | -5.0 ± 2.7 | -0.4 ± 3.2 | -3.4 ± 2.2 | 7.6 ± 5.5 | **< 0.001** |
|  |  | **AD-LM** | 16.1 ± 3.7 | 14.0 ± 3.6 | 16.7 ± 3.9 | 12.3 ± 4.0 | 16.0 ± 3.1 | 8.9 ± 3.5 | **< 0.001** |
|  |  | **PD-LM** | 2.3 ± 2.7 | -2.2 ± 3.0 | 2.3 ± 2.4 | -3.3 ± 3.4 | 3.4 ± 2.0 | -4.8 ± 4.2 | **< 0.001** |
|  |  | **API-LM** | 12.4 ± 3.5 | 13.0 ± 2.5 | 12.0 ± 2.6 | 13.0 ± 3.1 | 12.3 ± 3.2 | 12.3 ± 3.5 | 0.717 |
|  |  | **lPTT** | -7.4 ± 3.6 | -0.8 ± 3.5 | -9.8 ± 3.1 | 0.3 ± 3.8 | -9.3 ± 3.3 | 5.3 ± 3.6 | **< 0.001** |
|  | **Reader 2** | **AD-MM** | 6.0 ± 2.4 | 5.5 ± 2.2 | 7.5 ± 2.8 | 5.2 ± 3.3 | 7.3 ± 4.6 | 1.7 ± 2.5 | **< 0.001** |
|  |  | **PD-MM** | 2.3 ± 2.6 | 0.8 ± 3.5 | 3.6 ± 1.0 | 1.6 ± 2.6 | 2.3 ± 2.7 | -3.1 ± 5.7 | **< 0.001** |
|  |  | **API-MM** | 20.0 ± 5.5 | 19.0 ± 5.4 | 19.4 ± 4.7 | 18.0 ± 5.4 | 20.0 ± 6.2 | 19.3 ± 4.6 | 0.933 |
|  |  | **mPTT** | -4.4 ± 2.7 | -1.7 ± 3.3 | -5.4 ± 2.7 | -0.3 ± 3.3 | -3.8 ± 2.2 | 7.9 ± 5.2 | **< 0.001** |
|  |  | **AD-LM** | 16.1 ± 4.0 | 14.1 ± 3.4 | 16.7 ± 4.3 | 12.1 ± 4.2 | 15.7 ± 3.1 | 9.0 ± 3.4 | **< 0.001** |
|  |  | **PD-LM** | 2.3 ± 2.8 | -2.0 ± 3.3 | 2.2 ± 2.8 | -3.2 ± 3.1 | 3.8 ± 2.0 | -4.8 ± 4.5 | **< 0.001** |
|  |  | **API-LM** | 12.2 ± 3.2 | 13.7 ± 2.6 | 12.4 ± 2.3 | 13.2 ± 3.6 | 12.4 ± 3.1 | 12.6 ± 3.7 | 0.763 |
|  |  | **lPTT** | -7.7 ± 3.5 | -1.1 ± 3.4 | -10.0 ± 3.0 | 0.2 ± 3.6 | -9.4 ± 3.3 | 5.2 ± 3.4 | **< 0.001** |

**Supplementary Table 4: Assessment of Intra-Reader Reproducibility of Manually Registered Coordinates Used to Build Specimen-Specific 3D Joint Models.**

Mean inter-measurement deviations of registered coordinates of anatomic landmarks (a, b) and central bone axes of tibia (c, d) and femur (e, f). Data are presented as means ± standard deviation [mm]. All conditions and configurations of all specimens (n=11) were included.

| Coordinate | Definition | Mean ± SD [mm] |
| --- | --- | --- |
| a) | the tip of the cartilage-covered femoral trochlea | 0.50 ± 1.33 |
| b) | the centre of the tibial tuberosity | 2.19 ± 2.02 |
| c) | the central point of the most proximal tibial diaphysis | 0.69 ± 0.92 |
| d) | the central point of the most distal tibial diaphysis | 0.42 ± 0.72 |
| e) | the central point of the most proximal femoral diaphysis | 0.44 ± 0.83 |
| f) | the central point of the most distal femoral diaphysis | 0.44 ± 0.80 |

**Supplementary Text 1**

**Detailed Description of Post-Processing and Image Analysis**

- 1. **Manual Image Segmentations and Registration**

For each configuration (i.e., δ_0_ and δ_1_) and PCL-condition (PCL_intact_, PCL_partial_, and PCL_complete_), the bone contours of the tibia and femur were manually segmented on sagittal T1-weighted images by DK (medical student, 1 year of experience in musculoskeletal imaging) using ITK-SNAP imaging software (version 3.8.0, Cognitica, Philadelphia, PA, US).^44^ Additionally, anatomic landmarks and central bone axes were registered on segmentation outlines as coordinates on the Cartesian coordinate system using the integrated multi-viewer and line-and-ruler tools.^44^ Anatomic landmarks, central bone axes, and segmentation contours were reviewed and double-checked for consistency by LMW (5 years of experience in musculoskeletal imaging). For registration of anatomic landmarks and femoral and tibial central bone axes, the following coordinates were defined:

a) the most proximal extension of the femoral trochlea (i.e., its tip), which was still covered by articular cartilage,

b) the centre of the tibial tuberosity,

c) the central point of the most proximal tibial diaphysis,

d) the central point of the most distal tibial diaphysis,

e) the central point of the most proximal femoral diaphysis, and

f) the central point of the most distal femoral diaphysis.

Prior to implementation of the 3D motion analysis model, automatic pre-processing techniques were performed to ensure consistent segmentation outlines. Voxels inside the manual segmentation outlines, yet by mistake not segmented, were automatically included using a connected-component analysis. Correspondingly, voxels outside of the manual segmentation outlines, mistakenly segmented, were excluded. Resulting segmentation outlines were double-checked for consistency by LMW. All data was subsequently exported into Python (v3.7.3,, Python Software Foundation, Wilmington, Del, US).

- 1. **Image Post-Processing to Quantify Joint Laxity**

For multidimensional determination, quantification, and evaluation of knee joint motion in response to loading, additional parameters including computed fixpoints, vectors, and vector projections were determined prior to implementation. To this end, two fixpoints were automatically derived. More specifically, the point at which the femoral central bone axis (connecting coordinates e) and f)) intersects with the segmentation outlines, i.e., the articular surface, of the distal femur was determined as the femoral axis-surface-intersection (fASI). Correspondingly, the point at which the tibial central bone axis (connecting c) and d)) intersects with the segmentation outlines of the proximal tibia was defined as tibial axis-surface-intersection (tASI). Additionally, the two anatomic landmarks (i.e., coordinates a) and b)) were registered as femoral trochlea (FT) and tibial tuberosity (TT). These coordinates were imported into Python as ground data and used to implement a specimen-specific 3D motion analysis model.

**1.3 Multidimensional Quantification of Joint Laxity**

For quantification of knee joint laxity, Euclidean vectors were used to connect FT and TT (vector_FT) and fASI and tASI (vector_ASI) and the following measures were automatically derived for both vectors:

- vector magnitudes (“vector_FT”, “vector_ASI”) and

- vector projections on Cartesian x-axis (“x_FT”, “x_ASI”), y-axis (“y_FT”, “y_ASI”), and z-axis (“z_FT”, “z_ASI”). These measures served to quantify knee joint laxity in the three dimensions, i.e., in the anteroposterior, mediolateral, and craniocaudal dimension **(Supplementary Figure 1).**

Of note, for vector projections on the Cartesian x-axis, positive values imply that the femoral coordinate (FT, fASI) is located more laterally than the corresponding tibial coordinate (TT, tASI) with the vector passing from proximal-lateral to distal-medial. For vector projections on the Cartesian y-axis, positive values imply that FT or fASI are located more anterior than TT or tASI with the vector passing from proximal-posterior to distal-anterior. For vector projections on the Cartesian z-axis, positive values imply that FT or fASI are located more proximal than TT or tASI with the vector passing from proximal to distal.
